# Temperature-dependent development and survival of an invasive genotype of wheat curl mite, *Aceria tosichella*

**DOI:** 10.1007/s10493-021-00602-w

**Published:** 2021-03-04

**Authors:** Kamila Karpicka-Ignatowska, Alicja Laska, Brian G. Rector, Anna Skoracka, Lechosław Kuczyński

**Affiliations:** 1 Population Ecology Lab, Institute of Environmental Biology, Faculty of Biology, Adam Mickiewicz University, Uniwersytetu Poznańskiego 6, 61-614 Poznan, Poland; 2 USDA-ARS, Great Basin Rangelands Research Unit, Reno, NV USA

**Keywords:** Developmental time, Phytophagous mites, Survival, Temperature, Wheat curl mite

## Abstract

Quantifying basic biological data, such as the effects of variable temperatures on development and survival, is crucial to predicting and monitoring population growth rates of pest species, many of which are highly invasive. One of the most globally important pests of cereals is the eriophyoid wheat curl mite (WCM), *Aceria tosichella*, which is the primary vector of several plant viruses. The aim of this study was to evaluate temperature-dependent development and survival of WCM at a wide range of constant temperatures in the laboratory (17–33 °C). The development time of each stage depended significantly on temperature and it was negatively correlated with temperature increase. At high temperatures (27–33 °C), individuals had shorter developmental times, with the shortest (6 days) at 33 °C, whereas at the lowest tested temperatures (17–19 °C), developmental time was almost 3× longer. Moreover, temperature had a clear effect on survival: the higher the temperature, the lower the survival rate. These data provide information promoting more efficient and effective manipulation of WCM laboratory colonies, and further our understanding of the ramifications of temperature change on WCM physiology and implications for the growth and spread of this globally invasive pest.

## Introduction

Basic biological data, such as the effect of variable temperature on development, survival, and reproduction, are especially crucial for predicting and monitoring population growth rates of pest species, many of which are highly invasive. The increasing spread of non-indigenous or alien species to non-native regions is a globally important problem of increasing urgency (Dukes and Mooney 1999; Butchart et al. 2010; Hu et al. 2011; Cini et al. Cini et al., 2014; Seebens et al. 2017; Bianchi et al. 2019; Liu et al. 2020), as their spread is facilitated by the inexorable rise of globalization in travel and trade (Navia et al. 2010; Bertelsmeier et al. 2017; Vanbergen et al. 2018). The establishment of invasive species depends on their ability to reach new areas and the environmental conditions they encounter in new sites, including ambient temperatures. Environmental changes, such as elevated temperatures may increase the suitability of a given region to invaders from warmer climates (Walther et al. 2009). Increasing temperatures in particular may influence the development time and survival of pests and invasive species, allowing them to rapidly reach adult stages and produce offspring in a shorter time (Hanselmann et al. 2011; Salum et al. 2014; Ju et al. 2015, 2017; Xie et al. 2018). To address the issue of temporal and spatial dynamics and the invasive potential of non-indigenous species, it is critical to understand how temperature affects their development, survival, and other life-history traits.

Mites in the superfamily Eriophyoidea, numbering over 5000 species and characterized by their minute size (< 300 µm), relatively simple morphology, and obligate herbivory, are becoming widely recognized as globally adventive or invasive species (Navia et al. 2010). Their importance in the fields of agriculture and ecology has grown in the wake of recent significant advancements in microscopy and molecular biology that facilitate improved taxonomic (Navajas and Navia 2009; Monfreda et al. 2010; de Lillo et al. 2010; Chetverikov et al. 2015; Skoracka et al. 2015; Laska et al. 2018), behavioral (Skoracka et al. 2007; Michalska et al. 2010; Kiedrowicz et al. 2017; Laska et al. 2019), and other studies of these tiny, fascinating beasts. Many eriophyoid species have been recorded as agricultural pests, either due to their own feeding or as vectors of plant diseases (Navia et al. 2010; de Lillo et al. 2018). As such they have been employed as study subjects to test a variety of ecological hypotheses (Thomas and Hein 2003; Michalska et al. 2010; Navia et al. 2010; Oliveira-Hofman et al. 2015; Wosula et al. 2016; Kuczyński et al. 2016; Laska et al. 2017, 2019; Kiedrowicz et al. 2017). With this increased attention, there is a growing need for fundamental biological data from important eriophyoid species, such as data on their developmental responses to variations in ambient temperature and other abiotic factors that are essential to understanding their life histories and population dynamics.

One of the most globally important pests of cereals is the eriophyoid wheat curl mite (WCM), *Aceria tosichella*, which is the primary vector of *Wheat streak mosaic virus*, and several other plant viruses. It is a well-studied mite that is known to be a complex of cryptic species consisting of genotypes differing in several ecological traits (e.g., host plant range, thermal optima, ability to transmit viruses) (Skoracka et al. 2013, 2018a, b; McMechan et al. 2014; Kuczyński et al. 2016). The most pestiferous WCM biotypes (in Europe and South America known as MT-1 and MT-8, and in Australia and North America known as type 2 and type 1, respectively; Skoracka et al. 2018b) are the most widespread, occurring in major areas of cereal cultivation worldwide (North and South America, Africa, Europe, Asia, and Oceania) (Navia et al. 2013; Skoracka et al. 2014b). A positive correlation between temperature and population growth rate has been detected in these pestiferous biotypes, including intraspecific variation for this trait (Kuczyński et al. 2016). For example, the temperature ranges within which biotypes’s populations were able to increase were: 12.2–40.0 °C for MT-1 and 10.4–35.7 °C for MT-8, with the highest temperatures representing the upper thresholds for survival, and the optimal temperature for population growth were 35.1 and 31.9 °C for MT-1 and MT-8, respectively (Kuczyński et al. 2016).

The broad range of WCM’s thermal tolerance also improves its colonization and invasive potential (Navia et al. 2013; Kuczyński et al. 2016). Moreover, its small size enables WCM, like many other eriophyoids, to escape detection when infesting commodities and to disperse on light wind currents (Navia et al. 2013). Given the aforementioned and other characteristics (e.g., easy laboratory manipulation, relatively short generation time, and well-established laboratory rearing protocols), WCM represents a useful model species for the study of Eriophyoidea and other invasive arthropods; as such the benefits of accumulating basic biological data for WCM are ultimately multiplied.

Whereas some life-history parameters of WCM (sensu lato) have been observed under specific temperatures (e.g., egg development, mite survival) (del Rosario and Sill 1965; Boczek and Chyczewski 1975; Sabelis and Bruin 1996; Skare et al. 2003) there is currently lack of information on the survival and development time of specific WCM biotypes while inhabiting their hosts over a wide range of constant temperatures. Wosula et al. (2015) tested the effect of temperature on off-host survival of WCM biotypes 1 and 2 and showed that length of survival of both biotypes decreased with increasing temperature. Such data are essential for the development of phenological models and in the case of pestiferous biotypes, effective management plans and preventive strategies.

The primary goal of this study was a comprehensive assessment of the effects of temperature on ontogenetic development and survival of WCM genotype MT-1 (also known as type 2). Temperature-dependent development and survival was evaluated at a wide range of constant temperatures in the laboratory (17–33 °C) within its biological limits, which were tested previously (Kuczyński et al. 2016). These results were then used to establish optimal temperatures for rearing WCM under laboratory conditions and to determine the relationship between temperature and generation time. This information is extremely important for designing experiments involving this species and for long-term maintenance of laboratory colonies. Additionally, these results aid assessments of the invasive potential of WCM MT-1 under changing climatic conditions, and provide a starting point for analogous studies of other Eriophyoidea.

## Materials and methods

### Study system

The subject of the study was *A. tosichella*, genotype MT-1 (Skoracka et al. 2014a). This genotype was chosen due to its high invasive potential compared to other lineages within the WCM complex. The MT-1 lineage is distributed worldwide, having been recorded in the Nearctic, the Palearctic and the Australasian realms (Skoracka et al. 2014b). MT-1 inhabits wild grasses (Poaceae), including smooth brome, tall oat-grass, wall-barley, and quackgrass; and cultivated grasses, i.e., cereals, including wheat, triticale, barley. MT-1 can also survive on plant species in the family Amaryllidaceae (e.g., onion and garlic) (Skoracka et al. 2013).

A mite stock colony was maintained in the Population Ecology Lab, Faculty of Biology, Adam Mickiewicz University. WCM MT-1 specimens used to establish this stock colony were collected in July 2009 from wheat in Choryń, Poland (52.0433°, 16.7672°; GenBank acc. no. JF920077). The MT-1 genotype was identified by sequencing the mitochondrial cytochrome c oxidase subunit I gene fragment (COI) (Skoracka et al. 2013). All experimental animals were reared on bread wheat plants, *Triticum aestivum* L., variety ‘Muszelka’, grown in pots from commercially available seeds. Infested plants were kept in cages consisting of metal frames wrapped in fine nylon mesh (micron size 44) to avoid contamination, under laboratory conditions (22–24 °C, ca. 45% RH, L12:D12).

### Testing WCM developmental time and survival at various temperatures

To test WCM development and survival at various temperatures we used the Murashige and Skoog (MS) medium (Murashige and Skoog 1962) modified by Karpicka-Ignatowska et al. 2019. This is the only method allowing for monitoring daily eriophyid mites development. Due to WCM microscopic and hidden life style, it is not possible to monitor daily development of these mites on the whole plants. Modified MS medium consisted of a solution of basic chemical nutrients and phytohormones to maintain leaf fragment turgidity and prevent deterioration, prepared according to Karpicka-Ignatowska et al. (2019). We transferred single females of WCM from the stock colony to 5 × 5 mm wheat leaf fragments placed on modified MS medium. Due to the minute size of WCM, it may be difficult to determine the sex without examination under a phase-contrast microscope. However, females are generally longer (ca. 30%) than males and on this basis we chose females for this experiment. Afterwards, mites were incubated in growth chamber at 10 constant temperatures ranging from 17 to 33 °C, at 2-°C intervals, 80 ± 5% RH (the optimal humidity conditions for maintaining good condition of leaf fragments and WCM development according to preliminary observations) and L16:D8 photoperiod. There were 10 replications for each temperature treatment. Females were monitored daily and removed from the experimental arena after laying their first egg. Only individuals that laid eggs (thus females) were included in the experiment. Mite development was monitored daily and the time needed for reaching each stage was recorded (larva, quiescent larva, nymph, quiescent nymph, adult). Female’s progeny was incubated and controlled until the F1 adult deposited the first egg or until the observed individual died. The time of individuals’ death was recorded and included in the estimation of WCM survival for the given temperature treatment. A complete generation of mite development was defined as the time of oviposition by an experimental female until the first egg of the next generation. Females’ survival was assessed to their first oviposition. After the experiment, adult individuals were mounted on microscope slides in modified Berlese medium (Monfreda et al. 2010) and their sex was determined using a phase-contrast microscope (Olympus BX41).

### Statistical analysis

To test how temperature influences the development time of each WCM MT-1 stage, as well as cumulative development time (the entire life cycle: from egg to next generation egg), a generalized additive model (GAM) was used (Wood 2017). The Gamma distribution with the log-link function was used for the response variable.

An Additive Cox Proportional Hazard Model (Hastie and Tibshirani 1990; Wood et al. 2016; Wood 2017) was used to test the influence of temperature and sex on WCM survival. At first, two candidate models were generated: the interaction model, which included sex as a parametric term and smoothers for the temperature (fitted separately for each sex), and the main effects model, with a common smooth fit for both sexes. Then, candidate models were compared based on the Akaike information criterion (AIC), with the model having the lowest AIC value being considered the best. Statistical analysis was performed in R v.4.0 (R Core Team 2019) using the mgcv 1.8 package (Wood 2017).

## Results

**Fig. 1:**
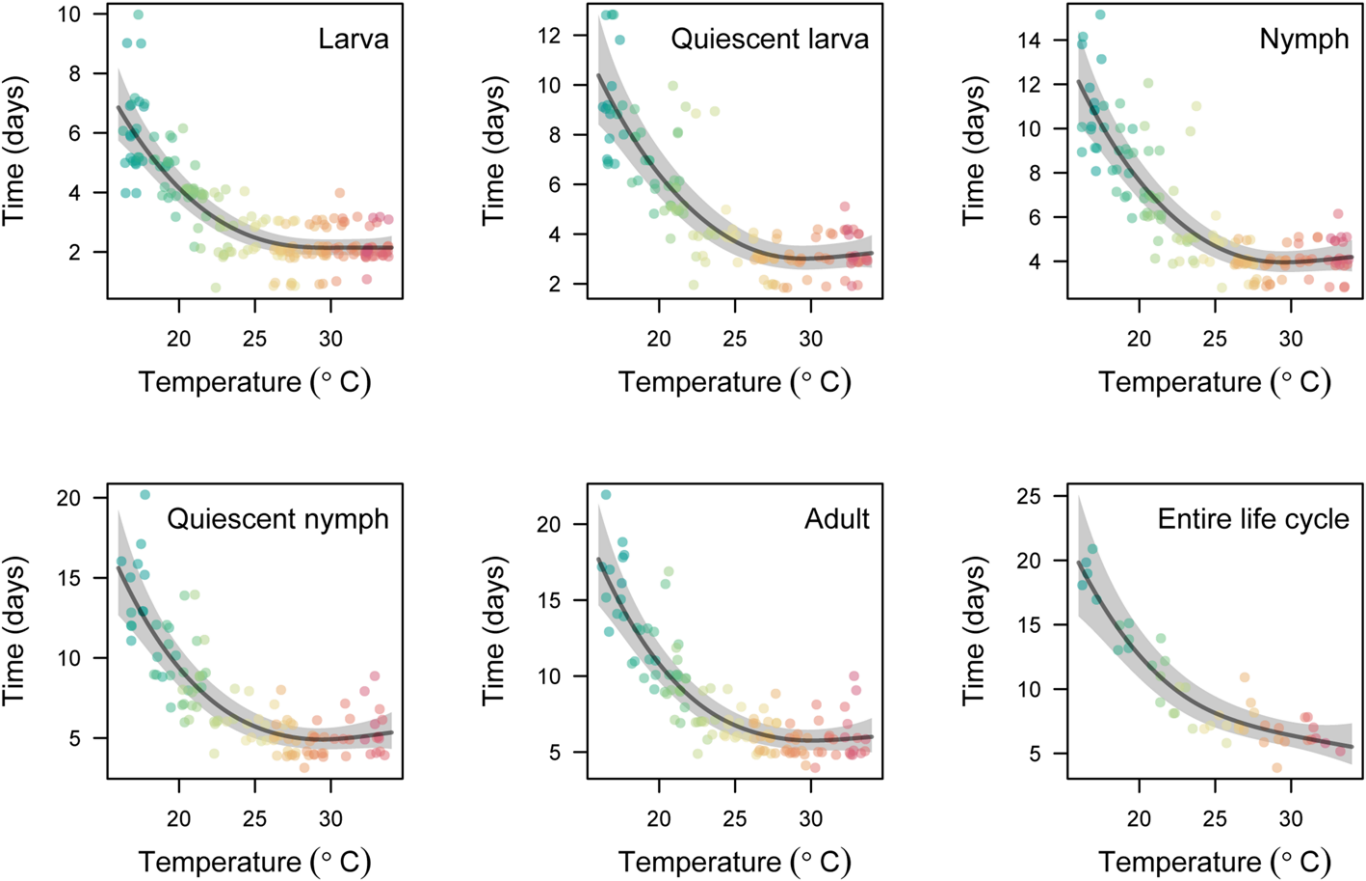
Wheat curl mite genotype MT-1 developmental time for all stages (cumulative days needed for reaching a given stage from an egg) in relation to the temperature. Solid lines are generalized additive model fits and shaded regions represent 95% confidence bands around these fits

WCM MT-1 showed successful egg-to-egg development across the whole range of temperatures tested, from 17 to 33 °C (Table 1, Fig. 1). The development time of each stage depended significantly on temperature (Table 1) and the general pattern of development was consistent during ontogeny (Fig. 1). The rate of development of WCM MT-1 individuals accelerated with increase in temperature. At high temperatures (27–33 °C), individuals had shorter developmental times, with the shortest (average < 6 days) at ca. 33 °C, whereas at the lowest tested temperatures (17–19 °C) egg-to-egg development time was almost 3 × longer (Fig. 1, Table 2). Mean cumulative development time ranged from 17.7 days at 17 °C to 5.7 days at 33 °C (Table 2).

**Table 1:** Parameters for the smoothing term in generalized additive models examining the relationship between temperature and development time for all stages of wheat curl mite genotype MT-1

Stage	Sample size (no. of individuals)	edf	F	p	Deviance explained (%)
Larva	188	2.51	128.5	< 0.0001	68.6
Quiescent larva	128	2.56	99.0	< 0.0001	74.2
Nymph	143	2.59	122.3	< 0.0001	77.2
Quiescent nymph	112	2.49	91.8	< 0.0001	76.1
Adult	122	2.52	108.9	< 0.0001	78.3
Entire cycle	45	1.97	85.2	< 0.0001	84.8

Estimated degrees of freedom (edf) reflect the smoothness of the fitted curve (a value of 1 represents a straight line)

**Table 2:** Entire life cycle development time at constant temperatures predicted from the generalized additive model

Temperature (°C)	Life cycle duration [days]	95% confidence intervals
17	17.7	15.7–19.9
18	15.8	14.3–17.3
19	14.1	14.3–17.3
20	12.6	11.7–13.6
21	11.4	10.6–12.3
22	10.3	9.6–11.1
23	9.4	8.8–10.2
24	8.7	8.1–9.4
25	8.1	7.6–8.8
26	7.7	7.1–8.2
27	7.3	6.8–7.8
28	6.9	6.5–7.5
29	6.7	6.2–7.2
30	6.4	5.9–6.9
31	6.2	5.6–6.8
32	5.9	5.3–6.7
33	5.7	5.0–6.6

**Fig. 2:**
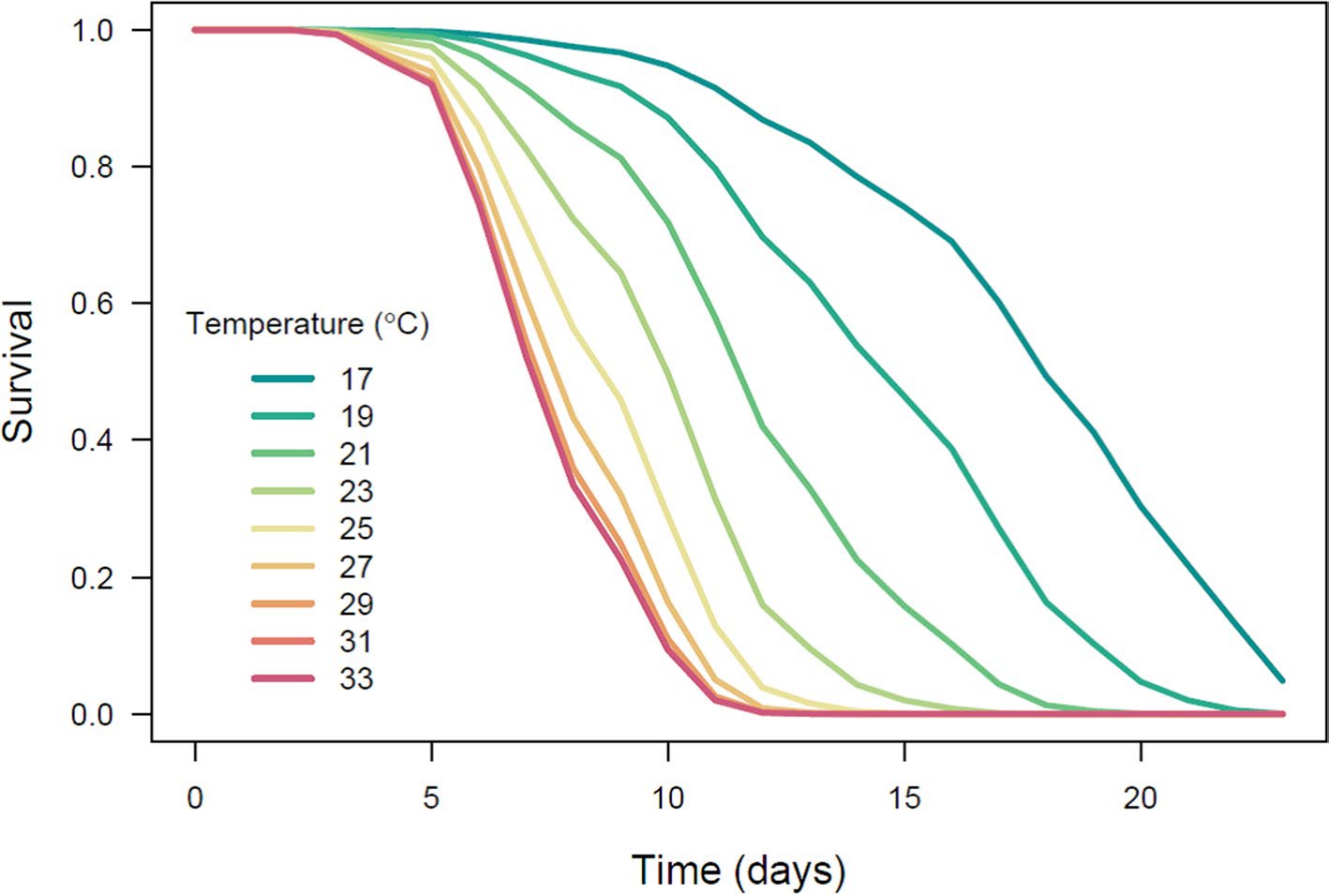
Survival curves for wheat curl mite at 17–33 °C

**Fig. 3:**
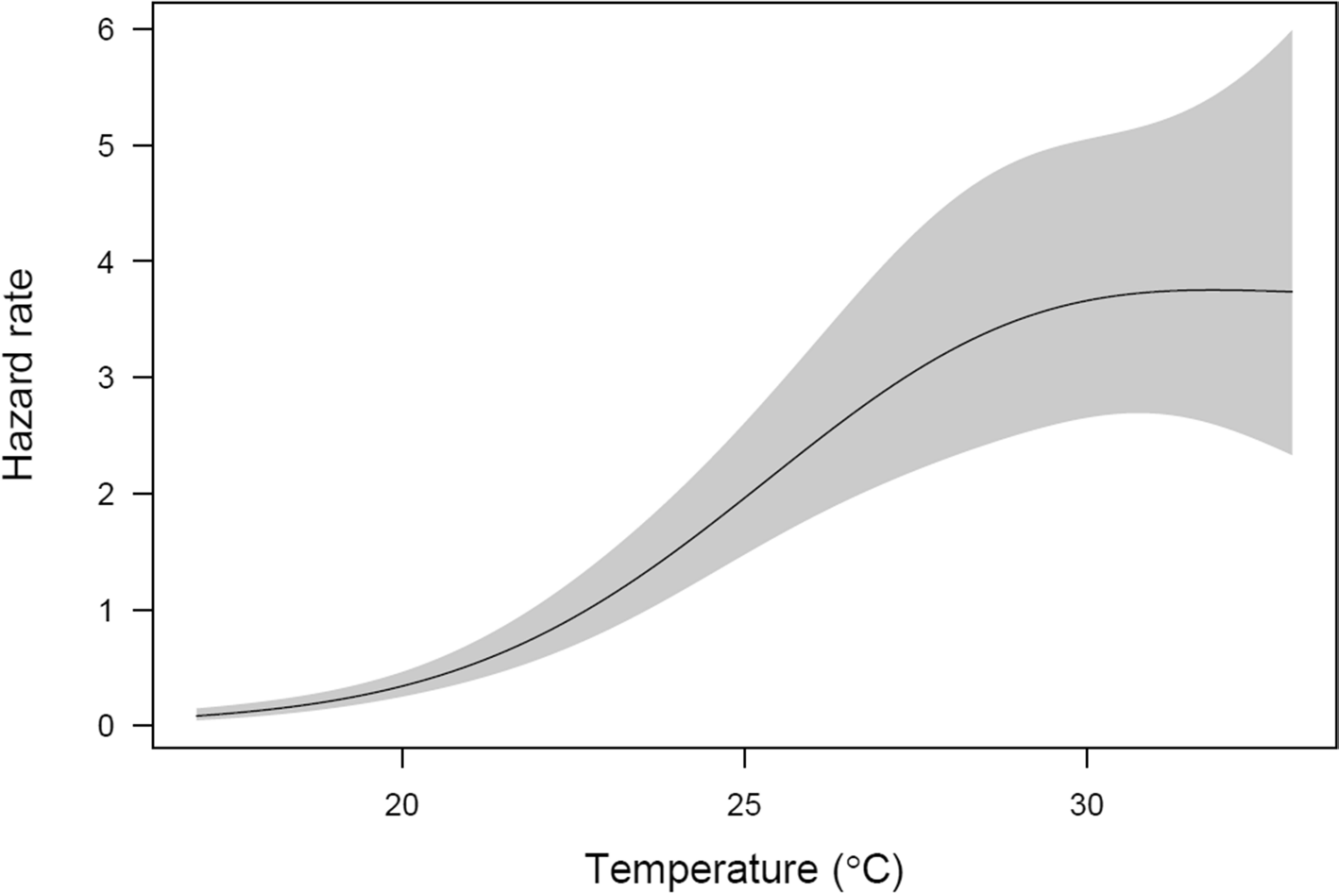
Relationship between development temperature (17–33 °C) and the hazard rate. The line represents the hazard rate for wheat curl mite estimated by the Additive Cox Proportional Hazard Model and the shaded region is the ± 2SE band

There were no signs that the pattern of temperature influence on hazard rate differed between the sexes (there was 6:1 female:male ratio, 85.7% females in the population). Based on AIC values, the main effects model was the best fit, given the data (main effects model AIC = 474.8 vs. interaction model AIC = 475.9). Moreover, in the main effects model, sex had no clear effect on a hazard rate (P = 0.84), suggesting that the sexes did not differ in their baseline hazards. Thus, in the final model, sex was excluded, and the only predictor remaining in this model was temperature, which had a clear effect on survival: the higher the temperature, the lower the survival rate (GAM results: λ^2^ = 84.5, edf = 2.4, p < 0.0001, n = 275; Fig. 2). However, the relationship between temperature and hazard rate is non-linear: the risk of death increases as the temperature rises, but above ca. 29 °C there is no further change (Fig. 3).

## Discussion

The accumulation of basic biological data represents an essential resource for comprehensive studies of species like wheat curl mite. In addition to being an economically important pest species, WCM is the de facto model for the study of Eriophyoidea, due to the relative ease with which it can be collected from the field, on at least four continents (Carew et al. 2009; Skoracka et al. 2012, 2014b, 2018a; Miller et al. 2013; Karpicka-Ignatowska et al. 2019; Khalaf et al. 2020), and with which it can be cultured in the laboratory (e.g., Karpicka-Ignatowska et al. 2019), as well as its well-documented genetic and physiological diversity (Carew et al. 2009; Hein et al. 2012; Navia et al. 2013; Szydło et al. 2015; Skoracka et al. 2018a). Such studies have yielded important data on passive dispersal in microscopic eukaryotes (Laska et al. 2019; Karpicka-Ignatowska et al. 2019), virus transmission by herbivorous arthropods (Seifers et al. 2002; Schiffer et al. 2009; Navia et al. 2013; McMechan and Hein 2015, 2017; Wosula et al. 2018; Singh et al. 2018; Tatineni and Hein 2018), physiology (Wosula et al. 2015), genetics (Miller et al. 2013; Skoracka et al. 2018a), ecology and behavior (Kiedrowicz et al. 2017; Laska et al. 2019), distribution (Schiffer et al. 2009; Navia et al. 2010, 2013; Skoracka et al. 2014b; Khalaf et al. 2020), as well as specific assessments of its impact on crop losses (Navia et al. 2013; McMechan et al. 2014; Singh et al. 2018; Skoracka et al. 2018b). In this study we have broadened the knowledge about this important plant parasite by providing basic information on the functional relationship between temperature and ontogenetic development and survival of WCM MT-1 biotype over a wide range of temperatures, comprising the first such report for this species.

The current research enables more effective manipulation of WCM laboratory colonies to streamline studies investigating the effects of specific mite life stages on host plants (e.g., dispersal, demographics, disease transmission), and furthers our understanding of the ramifications of temperature change on WCM physiology and implications for the growth and spread of this globally invasive pest and other non-model eriophyoid species. This enhanced knowledge also creates the possibility of manipulating colony developmental time, increasing or reducing the number of mite generations during a given period according to the goals of the project. For example, fewer generations may be desired for experimentally selected lines being reared in the laboratory, to reduce the incidence of random genetic mutations. By contrast, shorter generation times may be desired when the goal is to quickly produce a large number of individuals from a given population or in genetic studies of adaptation to environmental conditions imposed in the laboratory (e.g., experimental evolution, artificial selection). This precise information about temperature-dependent life-history traits informs planning for complex, long-term studies and should thus accelerate progress. In the field, such data enable improved estimation of intra-specific demographics for ecological and host-pest synchrony studies (Pulatov et al. 2016; Huang and Hao 2020). It is important to note that the study presented here was performed under laboratory conditions designed specifically to maintain minute phytophagous arthropods; in this case, mites were reared on plant fragments placed on an artificial medium. In this way, we were able to precisely estimate development and survival. It would be valuable in the future to validate these data under more natural conditions.

The negative correlation we found between temperature and development time (Table 2, Fig. 2) is a commonly observed phenomenon in invertebrates (Schwartzberg et al. 2014; Soroye et al. 2020). However, it adds substantial evidence to previous studies showing that the effect of rising temperature on the development and survival of WCM presents important agricultural and environmental challenges resulting from its accelerated development and invasion potential (Navia et al. 2013; Wosula et al. 2015; Kuczyński et al. 2016). As rising global temperatures exacerbate challenges posed by ectothermic pests (Cannon 1998; Bebber et al. 2013; Lehmann et al. 2020), information is needed to anticipate how these changes will affect specific taxa. Combining laboratory data, such as those presented here, with those collected in the field facilitates the creation of models to predict population growth rate and population dynamics under natural conditions (Fand et al. 2015; Kuczyński et al. 2016). As such, the improved fundamental understanding of thermal effects on phenology and survival of the model species WCM will enable the design of tools to predict mite outbreaks in the field and develop strategies for integrated pest management of WCM and other eriophyoid pests.

From the data presented here it is clear that the higher the temperature the shorter the developmental time of MT-1 (Fig. 1, Table 2), and that survival of MT-1 decreases as temperature rises, but above 29 °C the hazard rate does not change (Fig. 3). The shorter the developmental time, the faster adults start to reproduce. Interactions between these parameters (development, survival, and reproduction) determine the population growth rate. Although we did not measure reproduction directly, we can conclude that the shorter the time to sexual maturity, the sooner adult individuals will reproduce, thus population growth will be higher, provided that hazard rate will not substantially increase, as was shown in our results for temperatures above 29 °C. Shorter generation times, combined with higher proportions of individuals reproducing at higher temperature strongly suggest that rising temperature will result in increased population growth of MT-1 as temperature approaches the previously estimated optimum for MT-1 population growth of 35.1 °C (Kuczyński et al. 2016). Therefore, the implications for rising temperatures in cereal growing regions of the world are clear.

It is also important to note that the greatest gains in rate of WCM development are found at the lower end of the tested temperature range (see Table 2). For example, under a hypothetical scenario of a local rise of 4 °C, a relatively cool cereal-growing region (17–21 °C during the growing season) that warms to more moderate temperature (21–25 °C) would face a decrease in WCM development time of 28.9–35.6% (from a range of 11.4–17.7 to 8.1–11.4 days per generation), increasing the number of generations during a 30-day period from approximately 2–3 to 3–4 generations, with a concomitant increase in population size. By comparison, under a similar 4 °C rise, a region changing from a warm (25–29 °C) to hot (29–33 °C) climate would experience only a 14.9–17.3% decrease in WCM development time (from 6.7–8.1 to 5.7–6.7 days per generation); thus, whereas the development rate would increase, the number of generations expected in a 30-day period would remain roughly between four and five generations. Ergo, whereas warmer regions foster more generations of WCM than cooler regions, it can be postulated that there is great potential for increased WCM pest status in regions where temperatures would rise from cool to moderate levels. Increased pest status under warming climates has also been predicted for other ectothermic pest species (Pulatov et al. 2016; Gu et al. 2018; Iwamura et al. 2020) and the data presented here add to these global concerns.

## Data Availability

The datasets generated and analyzed during the current study are available in the Zenodo repository under: 10.5281/zenodo.4008542.

## Code availability

Custom code used in this study is available in the Zenodo repository under: 10.5281/zenodo.4008542.

## Declarations

### Research involving human and/or animal participants

This article does not contain any studies with human participants or animals performed by any of the authors.
